# Supplementary material for: Cardiac arrhythmias in hospitalized patients with COVID-19: A prospective observational study in the western United States
Source: PLoS One. 2020 Dec 28;15(12):e0244533. doi: 10.1371/journal.pone.0244533 (PMC7769280; doi:10.1371/journal.pone.0244533)
Supplement: Attachment — Submitted filename: Response to Reviewers.docx [file pone.0244533.s001.docx]

**Response to Reviewers**

Reviewer #1:

This is an interesting and potentially important manuscript which examines cardiac arrhythmias in patients who are hospitalized because of Covid 19. The study was limited to patients who were placed on telemetry. A total of 143 patients were enrolled, all of them from a single site. The overall mortality was 25.2%. The major conclusion from the telemetry was that the survivors were significantly less tachycardic than the patients who did not survive (mean heart rate 90.6 BMP vs. 99.3 BPM -- although both groups had an elevated heart rate compared to healthy relaxed human subjects (there was no healthy control group). PVC's were also significantly more common in the non-survivors. Significant differences were also found in three laboratory tests: The survivors had a significantly lower peak troponin (0.03 vs 0.18); a significantly lower C reactive protein (97 vs 181); and a significantly lower interleukin 6 value (30 vs 296). PVC's and non sustained ventricular tachycardia were infrequent with no difference between the groups. (This contrasts with some studies performed elsewhere). Atrial fibrillation occurred in 11.9% of patients and was not different between survivors and patients who died. There was one patient with asystole who died, one patient with pulseless electrical activity who died and one patient with ventricular tachycardia who survived. It is pointed out that all of the life threatening arrhythmias were in patients with an elevated troponin.

Another distinguishing feature of the survivors is that they had a higher body mass index, possibly because the non-survivors ate or drank less. The difference was statistically significant, but not drastically different.

The discussion contains reasonable explanations for the results, and notes the limitations of the study. No correlation could be demonstrated between anti-inflammatory markers and ventricular arrhythmias because the serious ventricular arrhythmias were infrequent. There were also no effects of drug treatment on arrhythmias, but the sample size was too small to rule this out. Remdesevir was administered in this study, but to a small fraction of the patients and there this study was not designed to determine its effect on overall clinical outcome.
The statistical comparisons were limited to non-survivors vs. survivors in a group of patients who were hospitalized and on telemetry. It would have been interesting to obtain similar data on patients who tested positive but were not hospitalized, or even healthy controls. There is certainly data on atrial fibrillation, PVC's and non-sustained VT in patients who were studied before 2019.

- We agree with the reviewer that it would be interesting to obtain data on COVID-19 patients who were not hospitalized and compare to patients who were hospitalized. However, it will be difficult to obtain data on rhythmic disturbances for COVID-19 patients who were not hospitalized. We can design a study to apply Holter monitor or ambulatory patch monitoring device (such as Zio patch) to non-hospitalized COVID-19 patients to monitor rhythmic disturbances, but such study will require separate design and IRB approval. It will be also interesting to compare our study to healthy controls. However, health controls are not admitted to hospital so it will be difficult to obtain telemetry data. Although they undergo ambulatory monitoring, it will be difficult to compare their ambulatory monitoring data to our study findings due to the heterogeneity of the population. The manuscript has now been edited to reflect this limitation in the discussion section.
- In Discussion (page 10, line 232-233)

Comparisons between COVID-19 patients who were hospitalized vs. non-hospitalized and even with healthy controls would have potentiated our findings.

It should also be mentioned that some patients with myocardial infarction have been avoiding hospitals because they fear exposure to covid 19, and that this has produced increased mortality. This should be mentioned with an appropriate reference. This paper does not conclusively establish why the patients who died had higher peak troponin levels, so it is possible that early hospital treatment could have been helpful. Also, as pointed out, there were no studies such as MRI or biopsy to confirm the presence of myocarditis.

- This is a really good point. We have modified the discussion and added the appropriate reference (Lancet, 2020) below.
- In Discussion (page 9, Line 221-224)

There is a possibility that the patients with coronary artery disease might have avoided seeing a doctor or coming to a hospital although they developed cardiac symptoms, due to the concerns of exposure to COVID-19 [13]. Such patients might have come to hospital eventually when they contracted COVID-19 and this could have led to high levels of troponin and high mortality.

- Reference (13)

Mesnier J, Cottin Y, Coste P, Ferrari E, Schiele F, Lemesle G, et al. Hospital admissions for acute myocardial infarction before and after lockdown according to regional prevalence of COVID-19 and patient profile in France: a registry study. Lancet Public Health. 2020;5(10):e536-e42. Epub 2020/09/21. doi: 10.1016/S2468-2667(20)30188-2. PubMed PMID: 32950075; PubMed Central PMCID: PMCPMC7498416.

Minor points: In figure 2 the three arrhythmias illustrated were presumably all obtained in different patients. This should be stated.

- These three rhythm strips were from 3 different patients. We have modified the figure legend as below.
- Figure 2. Malignant arrhythmias in three patients with COVID-19. A. Sustained polymorphic ventricular tachycardia. B. Polymorphic ventricular tachycardia degenerating into ventricular fibrillation. C. Complete AV block.

It could also be asked why the patients classified as DNR were kept on telemetry. Presumably patients sent to hospice are not on telemetry, but DNR patients are often sent non-acute wards with lower levels of nursing care.

- DNR patients are sometimes kept on telemetry because DNR does not mean that the patients are refusing active medical treatments. There are certainly benefits of telemetry in patients with DNR, such as monitoring heart rate in patients with atrial fibrillation or monitoring QT interval in patients with prolonged QT interval. When patients decided to withdraw care, then telemetry monitoring were taken off.
